# Risks of Stroke and Heart Disease Following Hysterectomy and Oophorectomy in Chinese Premenopausal Women

**DOI:** 10.1161/STROKEAHA.121.037305

**Published:** 2022-07-12

**Authors:** Michiel H.F. Poorthuis, Pang Yao, Yiping Chen, Yu Guo, Liya Shi, Liming Li, Zhengming Chen, Robert Clarke, Ling Yang

**Affiliations:** Clinical Trial Service Unit and Epidemiological Studies Unit (M.H.F.P., P.Y., Y.C., Z.C., R.C., L.Y.), Nuffield Department of Population Health, University of Oxford, United Kingdom.; Medical Research Council Population Health Research Unit (MRC PHRU) (Y.C., L.Y.), Nuffield Department of Population Health, University of Oxford, United Kingdom.; Fuwai Hospital, Chinese Academy of Medical Sciences, Beijing (Y.G.).; The First Affiliated Hospital of Hainan Medical College, Haikou, Hainan, China (L.S.).; Department of Epidemiology and Biostatistics, School of Public Health, Peking University Health Sciences Center, Beijing, China (L.L.).

**Keywords:** cardiovascular disease, china, heart disease, hysterectomy, women

## Abstract

Little is known about the long-term risks of stroke and ischemic heart disease (IHD) in women who had a hysterectomy alone (HA) or with bilateral oophorectomy (HBO) for benign diseases, particularly in China where the burden of cardiovascular diseases (CVD) is high. We assessed mean levels of cardiovascular risk factors and relative risks of stroke and IHD in Chinese women who had a HA or HBO.

**Methods:**

A total of 302 510 women, aged 30 to 79 years were enrolled in the China Kadoorie Biobank from 2004 to 2008 and followed up for a mean of 9.8 years. The analysis involved premenopausal women without prior cardiovascular disease or cancer at enrollment. We calculated adjusted hazard ratios for incident cases of CVD and their pathological types (ischemic stroke, hemorrhagic stroke, and IHD) after HA and HBO. Analyses were stratified by age and region and adjusted for levels of education, household income, smoking status, alcohol consumption, physical activity, body mass index, systolic blood pressure, diabetes, self-reported health, and number of pregnancies.

**Results:**

Among 282 722 eligible women, 8478 had HA, and 1360 had HBO. Women who had HA had 9% higher risk of CVD after HA (hazard ratio, 1.09 [95% CI, 1.06–1.12]) and 19% higher risk of CVD after HBO (1.19 [95% CI, 1.12–1.26]) compared with women who did not. Both HA and HBO were associated with higher risks of ischemic stroke and IHD but not with hemorrhagic stroke. The relative risks of CVD associated with HA and HBO were more extreme at younger age of surgery.

**Conclusions:**

Women who had either HA or HBO have higher risks of ischemic stroke and IHD, and these risks should be evaluated when discussing these interventions. Additional screening for risk factors for CVD should be considered in women following HA and HBO operations, especially if such operations are performed at younger age.

The incidence of cardiovascular disease (CVD) is lower in women than men, before the menopause, possibly reflecting the protective effects of ovarian hormones. Production of ovarian hormones declines substantially after oophorectomy, leading to a surgical menopause. The ovarian function is, however, also affected after hysterectomy alone (HA), possibly due to loss of collateral blood flow or paracrine or endocrine effects of the uterus on the ovaries.^1-3^ Such studies have prompted analyses of the long-term effects of HA or hysterectomy with bilateral oophorectomy (HBO) on risk of CVD.^4,5^

HBO is sometimes performed as a prophylactic procedure to reduce the risk of ovarian cancer in women having a hysterectomy for uterine leiomyomas, menorrhagia, metrorrhagia, pelvic organ prolapse, endometriosis, or pelvic inflammatory disease.^6-8^ The proportion of hysterectomies that include a prophylactic bilateral oophorectomy varies between countries, age, hysterectomy indications, and past medical or family history.^9,10^ About 40% of women undergoing hysterectomy have had a prophylactic oophorectomy in the United States, but little is known about corresponding rates in Chinese women.^11-14^

Previous studies had suggested that women who had HBO had higher risks of CVD, including stroke and ischemic heart disease (IHD).^15,16^ However, the results of such studies have been conflicting, as most such studies also included postmenopausal women at time of surgery. In addition, there is uncertainty about the risks of CVD after HA. Therefore, substantial uncertainty persists about the risks of CVD and CVD subtypes associated with HA or HBO in exclusive premenopausal women.

Little is known about the risks of CVD after HA and HBO in Chinese women where the burden of CVD, and particularly stroke, is higher than in Western countries.^17,18^ Hence, reliable assessment of the risks of CVD in operated women could result in appropriate targeting of treatment of modifiable vascular risk factors to reduce the incidence of CVD.^19^ The aims of the present study were to assess the risk of CVD and its component pathological types following HA and HBO in 2.8 million premenopausal Chinese women in the China Kadoorie Biobank.

## Methods

### Data Sharing

Anonymized baseline, resurvey, and cause-specific mortality and morbidity data are available for access through a formal application on China Kadoorie Biobank website (www.ckbiobank.org). The application will then be reviewed by a Data Access Committee. Further details about access policy and procedures can be found online at www.ckbiobank.org.

### Study Population

Details of the China Kadoorie Biobank study have been previously described.^20^ Briefly, 512 715 individuals (302 510 women and 210 205 men), aged 30 to 79 years were recruited between 2004 and 2008, from 5 urban and 5 rural areas in China. Trained health workers collected information on demographic, socioeconomic, and lifestyle characteristics, reproductive history, and medical history of each participant, using a laptop-based questionnaire (https://www.ckbiobank.org). Participants also had a range of physical measurements recorded, including blood pressure and various anthropometric measurements. A 10 mL blood sample was collected at baseline in all participants, and plasma levels of total cholesterol were measured in a subset of participants. Every 4 to 5 years, a random sample (5%–6%) of the survivors was resurveyed. All participants provided written informed consent for a protocol that had local, national, and international ethics approval.

### Women’s Reproductive History

Data on reproductive history included questions about age at first menstrual period, menopausal status (and age at menopause if postmenopausal), numbers of pregnancy and each pregnancy consequence (live birth, stillbirth, spontaneous or induced abortion), age at birth and length of breastfeeding for each live birth, and duration of use of oral contraceptive pills, and history of hysterectomy or oophorectomy (including age at surgery). Whether oophorectomy was unilateral or bilateral was determined by the reported age at oophorectomy and menopause. Bilateral oophorectomy was defined as participants who reported the same age of the operation and age at menopause, whereas unilateral was defined as the oophorectomy conducted before reported age at menopause.

### Follow-Up and Outcome Measures

Disease outcomes were ascertained during follow-up data linkage via a unique national identification number with national death registries, regional disease registries of major chronic diseases, and health insurance databases of all hospitalized events, in addition to annual active validation of survival and major chronic diseases using local residential, administrative, and medical records. Causes of death were classified by trained staff blinded to baseline information using the *International Statistical Classification of Diseases and Related Health Problems, Tenth Revision (ICD-10*). The main outcomes included in the present analyses were first events of CVD (*ICD-10*: I00–I09, I16–I25, I27–I88), IHD (*ICD-10*: I20–I25), including stroke (*ICD-10*: I60–I61, I63–I64) and stroke subtypes: ischemic (*ICD-10*: I63) and hemorrhagic (*ICD-10*: I61) stroke. By January 1, 2018, 18 434 (6.4%) women died and 3184 (1.1%) were lost to follow-up.

### Statistical Analyses

The study was performed according to STROBE guidelines (Strengthening the Reporting of Observational Studies in Epidemiology) for observational studies (Supplemental Material).^21^ Male participants (N=210 205) and women with a self-reported history of CVD (N=13 058) or cancer (N=1610) at baseline were excluded. Women who had oophorectomy without hysterectomy (N=2377) were postmenopausal at time of operation (N=697), underwent unilateral oophorectomy during hysterectomy (N=72), or with both operations at a different time (N=109) were further excluded (Figure). Analyses of responses to identical questions recorded at both baseline and resurvey questionnaires indicated that the accuracy of self-reported histories of hysterectomy and oophorectomy recorded at baseline and resurvey questionnaires was over 99% and responses to the baseline questionnaires were used for the present analyses.

**Figure.**
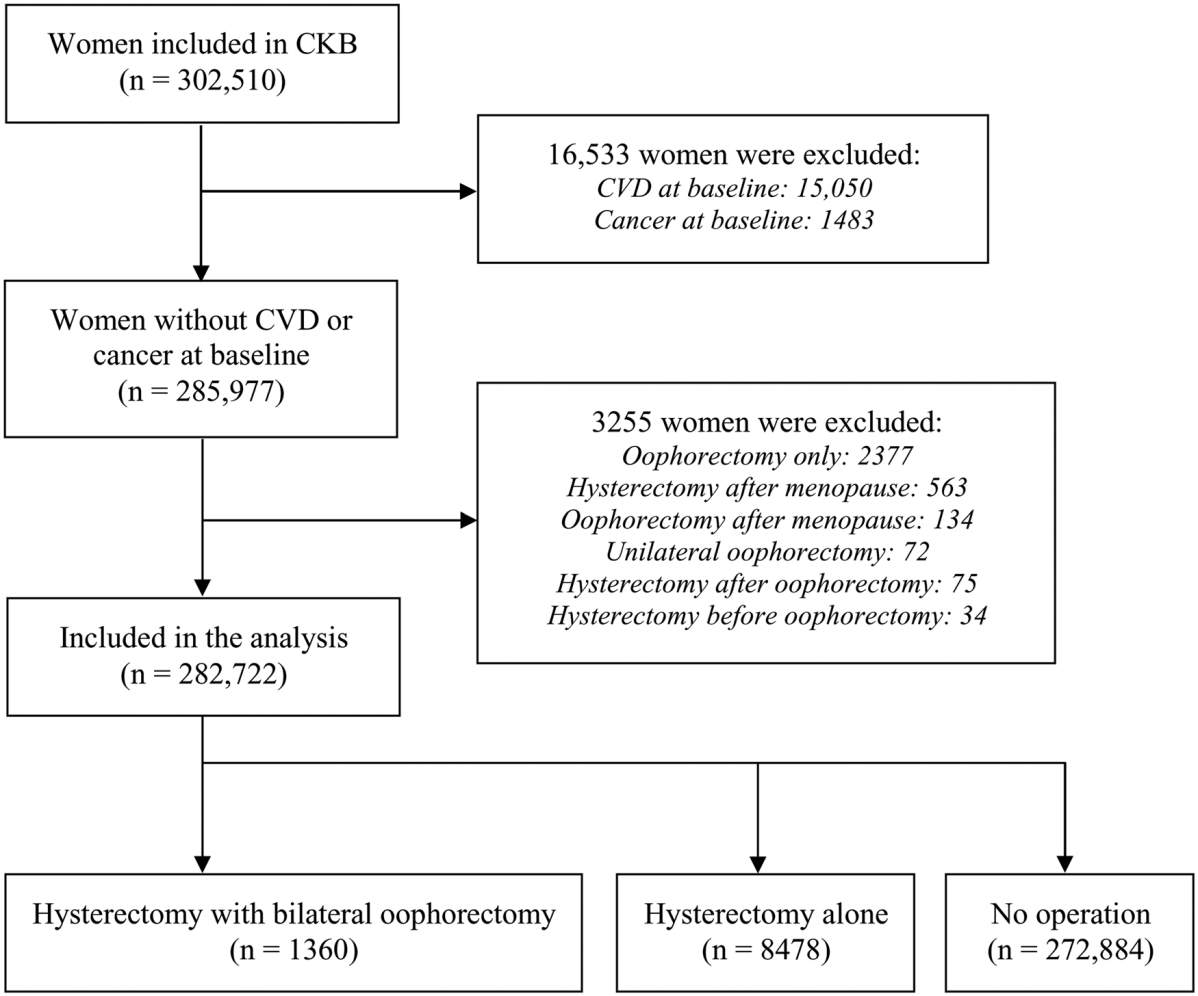
Flow diagram of participants included in the **China Kadoorie Biobank (CKB).** CVD indicates cardiovascular disease.

The prevalence and mean values of baseline characteristics by operation status at baseline were estimated using direct standardization to the age, sex, and area structure of the China Kadoorie Biobank population. Cox proportional hazards models were used to estimate hazard ratios for CVD, stroke, ischemic stroke, hemorrhagic stroke, and IHD in relation to HA and HBO, stratified by age at risk (in 5-year age groups) and region (10 groups), and adjusted for level of education (4 groups: no formal school; primary school; secondary school; ≥high school), annual household income (3 groups: <5000; 5000–20 000; ≥20 000 Chinese Yuan/y), smoking status (3 groups: never smoker; occasional/exregular smoker; smoker), alcohol use (3 groups: never regular drinker; exregular/occasional drinker; weekly drinker), physical activity (continuously assessed in metabolic equivalents of task h/day), body mass index (continuously assessed in kg/m^2^), systolic blood pressure (continuously assessed in mm Hg), diabetes (either self-reported or detected with blood tests at baseline) and self-reported poor health (yes/no; model 1). To minimize potential confounding by other reproductive factors, we also adjusted for number of pregnancies (model 2). The Cox proportional hazards assumption was checked using log cumulative hazard plots and the time-dependent coefficients in the Cox models. We also performed analyses by dividing the women according to age at surgery, using the overall mean age at menopause as cutoff (<48 versus ≥48 years). Group-specific confidence intervals were used for comparisons of more than 2 categories to allow comparisons of the different groups with each other without the choice of a reference group.^22,23^ All analyses were performed using R version 3.6.0.

## Results

Among the 282 722 women included in the present analyses, 9838 premenopausal women had a hysterectomy, of whom 8478 (86%) had HA and 1360 (14%) had HBO (Figure). Mean age at baseline questionnaire was 50.9 years for women without surgery, 51.9 for women who had HA, and 53.2 for women who had HBO. Mean age at surgery was 43.4 years for HA and 43.3 years for HBO. Compared with women without surgery, women who had HA were more likely to live in urban areas, were better educated, and reported a higher frequency of poor self-rated health. Compared with women without surgery, women who had HBO were older and more likely to be urban residents, have lower mean levels of blood pressure, body mass index, blood glucose, and HDL (high-density lipoprotein) cholesterol but higher mean levels of total and LDL (low-density lipoprotein) cholesterol and a slightly lower of number of pregnancies (Table 1).

**Table 1.**
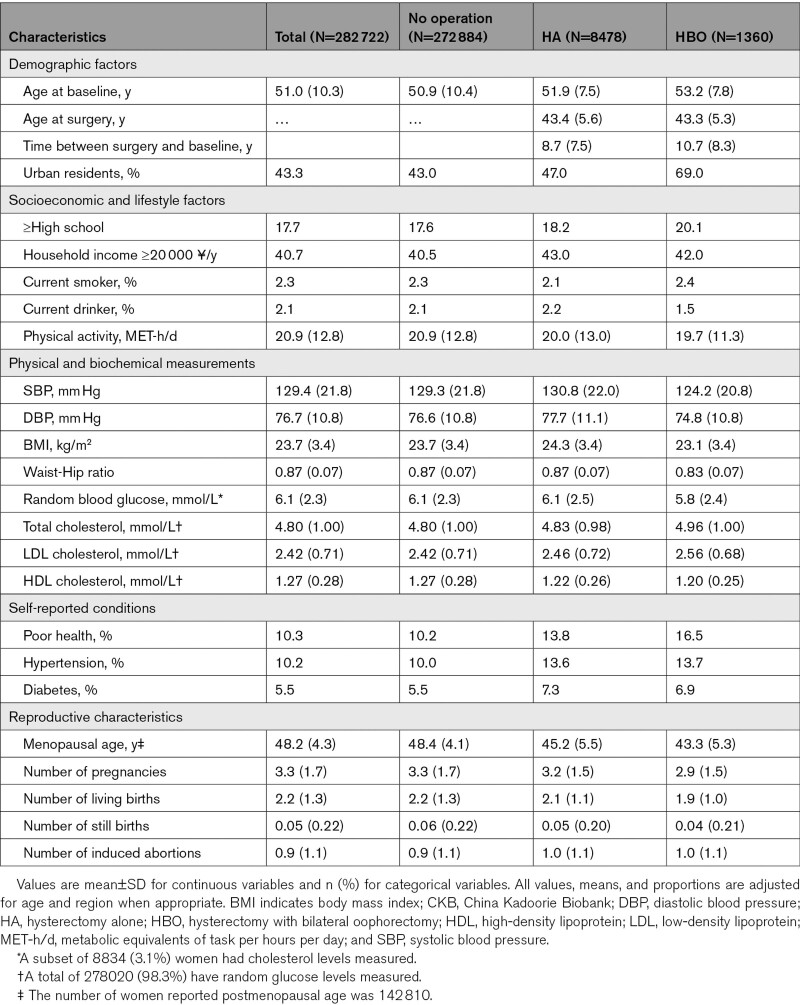
Baseline Characteristics of the Women in the CKB Study

Total cholesterol levels were similar after HA (4.83 mmol/L) and slightly increased after HBO (4.96 mmol/L) compared with women without surgery (4.80 mmol/L). LDL cholesterol levels were similar after HA (2.46 mmol/L) and slightly increased after HBO (2.56 mmol/L) compared with women without surgery (2.42 mmol/L).

During a mean follow-up of 9.8 (SD: 2.0) years, 68 288 women developed CVD (2358 occurring in the 8478 women who had HA and 467 in the 1360 women who had HBO). Of these, 25 075 women developed ischemic stroke (889 after HA and 210 after HBO) and 28 858 women IHD (1078 after HA and 231 after HBO).

Compared with women who had no operation, the adjusted hazard ratios for women who underwent HA were 1.09 (95% CI, 1.06–1.12) for CVD, 1.03 (95% CI, 0.99–1.08) for any stroke, 1.05 (95% CI, 1.01–1.10) for ischemic stroke, 1.11 (95% CI, 1.07–1.16) for IHD. In contrast, the adjusted hazard ratios for women who underwent HBO were 1.18 (95% CI, 1.11–1.25) for CVD, 1.18 (95% CI, 1.08–1.28) for any stroke, 1.19 (95% CI, 1.09–1.30) for ischemic stroke, and 1.17 (95% CI, 1.07–1.27) for IHD. However, women who had HA or HBO had no excess risks of hemorrhagic stroke (Table 2). These associations were unaltered by further adjustment for the number of pregnancies.

**Table 2.**
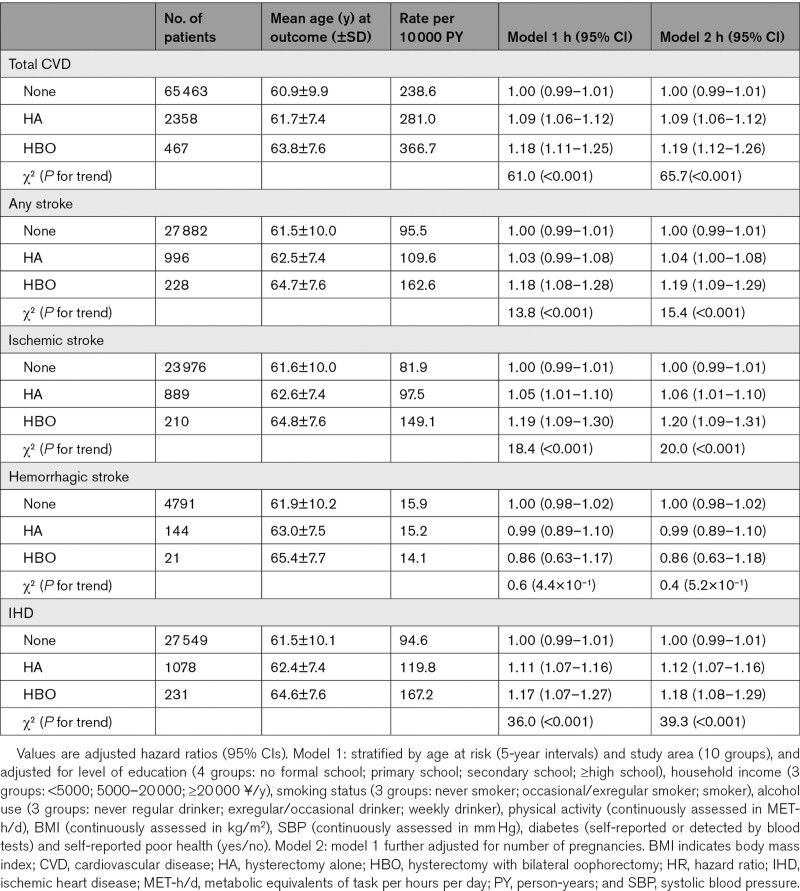
Adjusted Hazard Ratios for Risk of CVD With HA or HBO

Analysis by age of surgery indicated that the risks of CVD were higher in both younger and older women and with a significantly higher risks in women had their operation before age 48 years. Compared with women who did not have surgery, the hazard ratios for CVD among women who had HA before versus after age 48 years were 1.10 (95% CI, 1.06–1.13) versus 1.07 (95% CI, 1.02–1.13) and were 1.21 (95% CI, 1.13–1.30) versus 1.13 (95% CI, 1.01–1.28) among women who had HBO before versus after age 48 years, respectively. Similar findings were observed for risks of any stroke and ischemic stroke. In contrast, although higher risks were also observed for IHD in both age groups after HA, a significantly higher risk was observed in women who had HBO after age 48 years (Table 3).

**Table 3.**
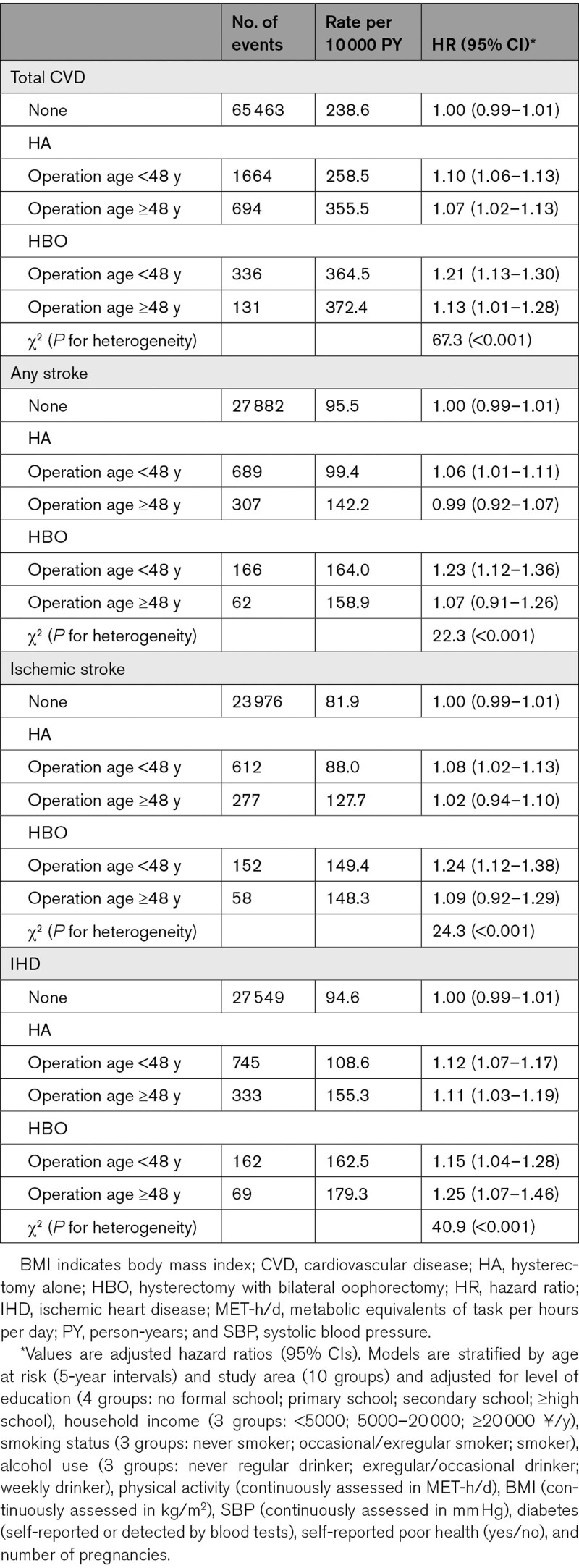
Risk of CVD With HA or HBO, by Age at Operation

## Discussion

In this prospective study of almost 300 000 women, women who had HBO had approximately 20% higher risks of CVD, ischemic stroke, and IHD than those without surgery. In contrast, women who had HA had 9% higher risks of total CVD, 6% higher risks of ischemic stroke, and 12% higher risk of IHD. These relative risks were more extreme at younger age of surgery.

Previous studies had reported no excess risk of stroke after HA, possibly due to the inclusion of both ischemic and hemorrhagic strokes in outcome measures.^24-26^ We demonstrated higher risks of ischemic stroke after HA but not hemorrhagic stroke. The results of the present study were consistent with those in UK Biobank, which also demonstrated increased relative risks of CVD after HA.^25^ Previous studies reported that HA alters ovarian function and women develop the menopause at an earlier age after HA.^1^ The reasons are not fully understood but may include impairment of the blood supply to the ovaries after HA.

The findings of the present study highlighted the importance of the strong decline of the production of ovarian hormones after HBO in premenopausal women. Other reasons for increased atherosclerotic CVD risk may include the effects of surgical menopause on increasing blood levels of total and LDL cholesterol. It is possible that the effects of HA and HBO on mean levels of blood lipids and blood pressure may accelerate risks of atherosclerotic diseases.^27-30^ Plasma levels of total cholesterol and LDL cholesterol in the present study were only slightly increased after HBO, possibly due to the relatively low age at surgery and short duration of follow-up.

Inclusion of postmenopausal women in previous analyses might have attenuated the relative risks of CVD following surgery. When risks were reported by age at operation, increased risks of CVD, stroke, and IHD were reported in younger women in the Swedish Inpatient Register.^31^ In that population-based cohort study using nationwide healthcare registers of over 0.8 million women, higher risks of CVD, stroke, and IHD were reported in women who were aged younger than 50 years at hysterectomy or at oophorectomy but not in women who were aged ≥50 years.^31^

### Strengths and Limitations

The present study had several strengths and limitations. This prospective study involved a large population with a complete and prolonged duration of follow-up. All women were premenopausal at time of operation. Women with CVD or cancer were excluded to minimize reverse causality bias.^32^ We calculated group-specific confidence intervals for each group, allowing for comparisons between HBO, HA, and no operation. We adjusted for not only established CVD risk factors but also characteristics that might influence hysterectomy rates, such as body mass index.^33^

However, data on the type of surgery and age at menopause were self-reported, and, for some women, data were collected several years after their menopause. Whether oophorectomy was unilateral or bilateral was determined by the self-reported age at oophorectomy and age at menopause because dates of surgery were not available. In addition, data on cardiovascular risk factors were measured after the operation and might be susceptible to recall bias or may have changed over time. Most women were relatively young to develop cardiovascular events, even with our prolonged duration of follow-up. Indications for hysterectomy were not recorded, but because participants with cancer were excluded from our analysis, we assumed that women underwent hysterectomy for benign indications.^24^ Women who were considered unsuitable for surgery were included in the no operation group, possibly underestimating the risks associated with HA and HBO. No data were available on *BRCA1* or *BRCA2* gene mutations, which might have influenced the decision to perform bilateral oophorectomy at the time of hysterectomy. Likewise, no data were collected on the use of hormone replacement therapy after surgery, but the use of hormone replacement therapy is typically low in China (<1%).^34^

### Implications for Clinical Practice and Future Research

The benefits of HA should be carefully balanced against nonsurgical treatment because HA increases the risk of CVD. Expert groups have advocated avoidance of prophylactic bilateral oophorectomy but encouraged bilateral salpingectomy to reduce the risks while maintaining premenopausal status.^35^ The results of the present study suggest that women who have undergone HBO, but also HA, should be considered for more intensive screening for CVD risk factors, particularly in younger women. Moreover, treatment of these women at high risk of CVD following HA and HBO with hormone replacement therapy may reduce the risks of atherosclerotic disease due to early loss of endogenous hormones.^36^

### Conclusions

Premenopausal women who had HA or HBO had higher risks of CVD, including ischemic stroke, and IHD. Women who were younger than 48 years at time of surgery had higher risks of CVD and ischemic stroke compared with those who had surgery at older age. More intensive cardiovascular risk management is indicated in such women. These risks should also be considered when advising women on the risks of surgery, particularly when deciding whether to remove or conserve the ovaries in women who require a hysterectomy.

## Article Information

### Acknowledgments

Drs Poorthuis, Yao, and Yang contributed to study concept and design. All authors contributed to acquisition, analysis or interpretation of data, and critical revision of the article for important intellectual content. Dr Yao contributed to statistical analysis. Dr Poorthuis contributed to drafting of the article. Dr Yang and R. Clarke contributed to study supervision.

### Sources of Funding

The China Kadoorie Biobank study is jointly coordinated by the University of Oxford and the Chinese Academy of Medical Sciences. The funding body for the baseline survey was the Kadoorie Charitable Foundation, Hong Kong, China and the funding sources for the long-term continuation of the study include UK Wellcome Trust (202922/Z/16/Z, 104085/Z/14/Z, 088158/Z/09/Z), Chinese National Natural Science Foundation (81390540, 81390541, 81390544), and the National Key Research and Development Program of China (2016YFC0900500, 2016YFC0900501, 2016YFC0900504, 2016YFC1303904). Core funding was provided to the CTSU, University of Oxford by the British Heart Foundation, the UK Medical Research Council, and Cancer Research UK.

### Disclosures

None.

### Supplemental Material

STROBE Checklist

Appendix

## Supplementary Material


